# The COVID-19 pandemic’s impact on psychotropic medication prescriptions for children and adolescents: a scoping review of quantitative longitudinal studies

**DOI:** 10.1186/s12991-026-00692-z

**Published:** 2026-08-17

**Authors:** Ida Aktner, Hilma Forsman, Viviane S. Straatmann

**Affiliations:** 1 Department of Public Health Sciences, Stockholm University & Centre for Health and Equity Studies (CHESS), Stockholm, Sweden; 2 Department of Social Work, Stockholm University, Stockholm, Sweden

**Keywords:** Scoping review, COVID-19 pandemic, Children, Adolescents, Psychotropic drugs, Mental health, Longitudinal studies

## Abstract

Global evidence shows a deterioration in the mental health of children and adolescents during the COVID-19 pandemic, raising questions about how mental health care, including psychotropic medication prescriptions, has adapted. However, it remains unclear how this deterioration affected prescription trends. This scoping review examined quantitative longitudinal studies comparing psychotropic drug prescriptions before and during the pandemic. A search of five databases, PubMed, CINAHL, Web of Science, Scopus, and PsycINFO, yielded 14 studies from 11 OECD countries (Austria, Australia, Canada, Denmark, Israel, Italy, the United States, Portugal, Sweden, Norway, and New Zealand). Included studies were quantitative, longitudinal, in English, and focused on individuals aged 0–19. Most used national health registers or clinical data covering pre-pandemic (2013–2019) and pandemic years (2020–2022). Six studies focused exclusively on adolescents (10–19 or 12–18 years), while others included larger age ranges (0–17/18/19 years). Ten studies reported increases in antidepressant and antipsychotic prescriptions during the pandemic, with two showing initial declines followed by rises; four reported overall decreases. Notable gender and age differences emerged, with girls showing the largest rise in antidepressant use and adolescents exhibiting higher usage than younger children. Stimulant prescription trends varied across countries. Socioeconomic disparities and national policy responses, including school closures and healthcare access, also influenced prescribing patterns. Two studies found higher prescription rates among children from more advantaged backgrounds. Twelve studies focused on the early pandemic period, limiting time generalisability, while only two extended into 2022. This review highlights the need for enhanced monitoring and extended longitudinal research to assess the long-term impact of crises like COVID-19 on youth mental health care. It also emphasises the importance of health system preparedness for managing psychiatric treatment in future public health emergencies. Although an overall shift in psychotropic prescribing among children and adolescents is suggested, the limited number of studies and short follow-up periods restrict conclusions about long-term trends.

**Supplementary Information:**

The online version contains supplementary material available at 10.1186/s12991-026-00692-z.

## Introduction

During the last decade, mental health problems among children and adolescents have grown into a significant public health concern worldwide [1]. This trend is evidenced by an increase in self-reported mental health concerns and rising prevalence of diagnosed disorders, such as depression and behavioural conditions. Moreover, these conditions have become leading causes of disease and disability among adolescents [1]. This increase in mental health diagnoses is reflected in a rise in healthcare utilisation and in the growing number of prescriptions for psychotropic drugs [1, 2]. Additionally, prescribing trends may indicate how treatments are implemented in practice [2], although they may at times not accurately reflect the actual needs of the group [3]. Previous studies suggest that the use of antipsychotics, antidepressants, and stimulants has surged globally among children and adolescents over the past decade.

The World Health Organisation (WHO) declared COVID-19 a pandemic in March 2020 and announced it was over in May 2023 [4]. The COVID-19 pandemic has exacerbated the already significant mental health challenges faced by children and adolescents globally [5–8], especially those with pre-existing mental health conditions, who faced notable psychosocial challenges during this period [8, 9]. The spread of the virus and the measures taken differed significantly across countries worldwide [4]. Some countries adopted prolonged lockdowns and isolation, while others preferred less restrictive interventions to mitigate potential degradative psychosocial consequences [10]. Access to health care treatment and assistance was strongly affected during the pandemic, regardless of the severity of the restrictions adopted [8]. Numerous appointments with healthcare providers were initially cancelled; video-call appointments became acceptable over time. Prescription renewals or dosage adjustments were sometimes conducted remotely, without in-person consultations with healthcare providers [5, 11].

The outlined situation indicates that the accessibility and continuity of mental health treatment were impacted as the pandemic evolved [8]. The existing reviews indicate a general rise in the frequency of prescriptions within the overall population, primarily focusing on adults during the pandemic [12, 13]. Although some studies have explored patterns of psychotropic medication prescriptions among children and adolescents during this period, a comprehensive synthesis of this evidence has not yet been conducted to offer an extensive understanding of the domain [5, 12]. Therefore, reviewing the existing literature on this topic could provide a better understanding of the scope and nature of the case for this specific group [14].

## Aim of the review

This study aims to review and synthesise the existing quantitative literature on the prescriptions of psychotropic drugs among children and adolescents (aged 0–19) during the COVID-19 pandemic. To address the aim, three research questions were raised:

I. What overall changes have taken place in the prescribing habits of psychotropic medications before and during the COVID-19 pandemic?
II. How did prescribing patterns vary by psychotropic drug type during the COVID-19 pandemic in comparison to the period before it?
III. In what ways did changes in psychotropic medication prescriptions during the COVID-19 pandemic vary by age, gender, and family socioeconomic status?

### Methods

A scoping review was considered the preferred approach, as it focuses on synthesising and mapping the existing literature and identifying potential knowledge gaps. This method is appropriate when research questions are more exploratory and the body of literature is heterogeneous in its methods [14]. The scoping review protocol followed the methodological framework developed by Levac et al. [15] and Arksey and O´Malley [16] and the checklist from the Preferred Reporting Items for Systematic Reviews and Meta-Analyses (PRISMA) Extension for Scoping Reviews [17].

## Search strategy

Five databases were searched: PubMed, CINAHL, Web of Science, Scopus, and PsycINFO, ensuring comprehensive coverage of the relevant literature. A block search combined three concepts: *Children and adolescents*,* Covid-19 pandemic*,* and Psychotropic drugs (i.e.*,* antidepressants*,* anxiolytics*,* hypnotics*,* stimulants*,* and antipsychotics*). The search incorporated MeSH terms and keywords based on the ‘Population, Concept & Context’ (PCC) framework. Articles required at least one term from each block, combined with “OR” within blocks and “AND” between blocks. Detailed search terms are provided in the Supplementary material.

## Inclusion and exclusion criteria

The eligibility criteria for the included studies were as follows: published between 2019 and 2024; covering at least one pre- and one post-pandemic time point; and referring to time points for data collection. The population of interest consisted of children and adolescents aged 0–19, according to the WHO’s age classification (0–12 years for children, 10–19 years for adolescents) [1]. Studies with broader age ranges were accepted if they reported results stratified for 0-19-year-olds. The included psychotropic drug categories are antidepressants (N06AB, N06A), anxiolytics (N05B), hypnotics (N05C), Stimulants (ADHD medication) (N06BA), and antipsychotics (N05A), as defined by the Anatomical Therapeutic Chemical Classification System (ATC) for pharmaceuticals [18]. Only peer-reviewed, English-language studies from the (Organisation for Economic Co-operation and Development (OECD) countries were included using quantitative and longitudinal designs. Studies from OECD member countries were included, as these countries have similar political and economic goals and are thus easier to compare and analyse [19]. Studies had to report pre- and post-pandemic prescription data to assess potential changes in prescribing patterns. Here, we refer to ‘post-pandemic’ as any time after the official beginning of the COVID-19 pandemic.

## Study selection process

**Fig. 1:**
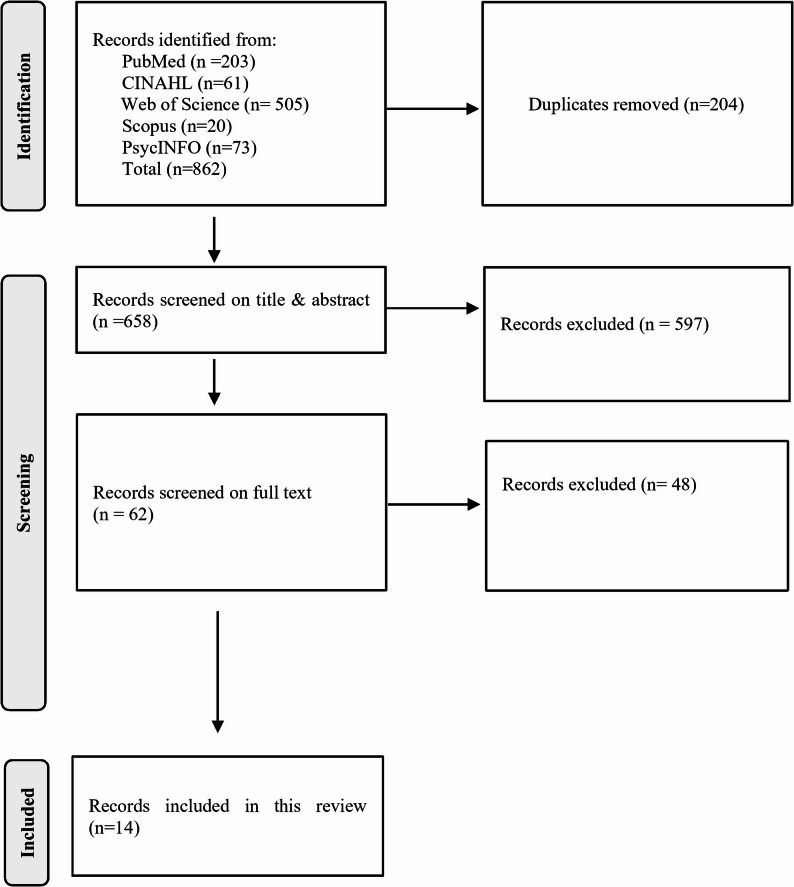
Prisma Flow diagram of the screening process

862 records were imported into the bibliographic software *Rayyan.ai*, with 204 duplicates removed. Two independent reviewers (I.A. and A.T.) screened the remaining 658 titles and abstracts, excluding 597. Of the 62 full texts assessed, 14 met the inclusion criteria (see Fig. 1).

## Data extraction

Relevant data were extracted from the included studies and organised in an Excel file by one reviewer (I.A.). Key information included: author and publication year, country, study population, sample size, study design and year, data source, statistical analysis, type of psychotropic drug, and data before and during the COVID-19 pandemic, including frequency of change and main findings. The extracted data were summarised into categories (see Table 1) and then synthesised into three themes in line with this review’s research questions. A table summarising all key information extracted from the included studies is described in the supplementary information.

**Table 1:** Characteristics of included literature

Authors and publicationyear	Country	Study population	Sample size	Study design and period	Data source	Statistical analysis	Type of drugs	Results	Main Findings
Antoniou et al. 2023	Canada	0 to 18 (0–13, 14–18)	All residents of Ontario, Canada, during the years the study was conducted	Longitudinal 2014–2022	Register data at the regional level	Interrupted time series analysis	Antidepressants, antipsychotics	21,4% increase in antidepressants 13,2% antipsychotics	There was an initial decrease in antidepressant prescriptions and then an increase in both psychotropics in the second wave (2021–2022) during the pandemic period compared to the pre-pandemic period (2014–2020).
Bilu et al. 2023	Israel	12 to 17	*N* = 200,000	Longitudinal study 2017–2021	Administrative clinical data at the individual level	Logistic regression	Antidepressants, anxiolytics, antipsychotics, stimulants	25% increase in antidepressants, 28% increase in antipsychotics, 10% decrease in stimulants	Increase in antidepressants and antipsychotics, decrease in stimulants during the pandemic (2020–2021) compared to the pre-pandemic period (2017–2019).
Bliddal et al. 2023	Denmark	5 to 17 (5–11, 12–17)	All residents of Denmark during the years the study was conducted	Longitudinal 2017–2022	Register data at the national level	Poisson regression	Antipsychotics, antidepressants, anxiolytics, hypnotics	Increased by 39% of hypnotics, 13% of antidepressants and 19% of stimulants	Increase in all psychotropic drugs during the pandemic period (2020–2022) compared to the pre-pandemic period (2017–2020).
Daneshmands et al. 2023	Canada	10 to 19 (10–14, 15–19)	*N* = 3974	Longitudinal 2018–2021	Administrative clinical data at the individual level	Interrupted time series analysis, least square and linear regression	Antidepressants	33,5% increase in antidepressants	Increase in antidepressants for children and school-age children during the pandemic (2020–2021) compared to the pre-pandemic period (2018–2020).
De Oliveira Costa et al. 2022	Australia	10 to 17	All residents of Australia during the years the study was conducted	Longitudinal 2015–2021	Administrative clinical data at the individual level	Interrupted time series analysis	Antidepressants	Increase of 56,9% in new use of antidepressants for girls and 45,75 for boys. Prevalent use: increase of 27,2% for girls and 34% for boys	Increase in antidepressants during the pandemic period (2020–2021), with a higher increase in new use among girls than boys compared to the pre-pandemic period (2015–2019).
Estrela et al. 2021	Portugal	0 to 17 (0–7, 8–17)	All residents of Portugal during the years the study was conducted	Longitudinal 2018–2021	National register data	Interrupted time series analysis	Antidepressants, anxiolytics, hypnotics	A decrease of 50% of antidepressants, hypnotics and anxiolytics	Overall reduction of antidepressants and anxiolytics, with a higher decrease of antidepressants during the pandemic period (2020- beginning of 2021) compared to the pre-pandemic period 2018–2020).
Kim et al. 2023	U. S	0 to 18 (0–12, 13–18)	All residents of Massachusetts, USA, during the years the study was conducted	Longitudinal 2011–2021	Aggregated register data at the regional level	Interrupted time series analysis	Stimulants	A decrease of 18.4% of stimulants among boys and 13% among girls	Decreased stimulant prescriptions during the pandemic period (2020–2021), with a higher decrease for boys than girls compared to the pre-pandemic period (2011–2019).
Ong & Roberts, 2022	Australia	12 to 18	*N* = 5960	Longitudinal 2019–2021	Administrative clinical data at the individual level	Logistic regression	Antipsychotics, antidepressants, stimulants	48,4% decrease of new prescriptions and 15,1% of follow-up prescriptions	Decrease in prescriptions of antipsychotics, antidepressants and stimulants, including new and follow-up prescriptions during pandemic period (2020–2021) compared to the pre-pandemic period (2019–2020).
Otter et al. 2024	Austria	10 to 19 (10–14, 15–19)	*N* = 850,000	Longitudinal 2013–2021	Register data	Interrupted time series analyses	Antidepressants, antipsychotics	Antidepressants: Age 15 to 19: Increase of 1.49% for females Increase of 2.04% for males Age 10 to 14: Increase of 104% compared to 35% among boys aged 10 to 14 years old. Antipsychotics: Increase of 5.38% for females Increase of 5.61% for males	Increase in prescriptions of antidepressants and antipsychotics among adolescents, particularly pronounced in girls aged 10–14 during the pandemic period (2020–2021) compared to the pre-pandemic period (2013–2020).
Pedersen et al. 2023	Norway Sweden Italy	0 to 17 (0–5, 6–11, 12–17) 0–14 in Italy	*N* = 3,455,521 (Total) *N* = 136,188 Italy *N* = 1,160,431 Norway *N* = 2,158,902 Sweden	Longitudinal 2018–2021	National and regional register data	Interrupted time series analysis	Antidepressants, antipsychotics, anxiolytics, hypnotics, stimulants	A decrease of 2.5 per 1000 stimulant prescriptions, a decrease of 0.7 per 1000 antidepressant prescriptions and a decrease of 0.6 per 1000 prescriptions of anxiolytics	Initial decrease in all prescriptions and then an increase in antidepressants, stimulants and anxiolytics, primarily in Sweden and Norway, during the pandemic period (2020–2021) compared to the pre-pandemic period (2018–2020).
Stephenson et al. 2022	Canada	10 to 18	*N* = 25,695 (7,96% of total)	Longitudinal study 2018–2021	Administrative clinical data at the individual level	Regression models (Generalised least squares, maximum likelihood).	Antidepressants	Overall, a 44% decrease in antidepressants	Decreased antidepressant use during the pandemic period (2020–2021) compared to the pre-pandemic period (2018–2020).
Tiger et al. 2024	Sweden, Norway, Denmark	0 to 19	Denmark *n* = 1,309,228 Norway *n* = 1,259,752 Sweden *n* = 2,289,467 (2015–2019) Denmark *n* = 1,296,400 Norway *n* = 1,248,749 Sweden *n* = 2,403,730 (2020)	Longitudinal study 2015–2021	National register data	Linear regression, ANCOVA	Antidepressants, hypnotics, anxiolytics	Sweden; 21,4% increase of antidepressants, 9,5% increase of antipsychotics Norway: 17,4% increase of antidepressants Denmark: 31,3% increase in antidepressants	Increase in antidepressants and antipsychotics during the pandemic period (2020–2021) compared to the pre-pandemic period (2015–2019.
Wood et al. 2023	Australia	0 to 18 (0–6, 7–12, 13–18)	*N* = 32,351	Longitudinal 2013–2021	Administrative clinical data at the individual level	Regression models (Linear regression)	Antidepressants, antipsychotics, anxiolytics, hypnotics, stimulants	Antidepressants: Boys: Increased by 6.1% Girls: Increased by 22.2% Stimulants: Boys: Increased by 14.5% Girls: Increased by 27.7%	Primarily, there was an increase in antidepressants and stimulants during the pandemic period (2020–2021) compared to the pre-pandemic period (2013–2020).
Zandy et al. 2023	Canada	0 to 19 (0–5, 5–9, 10–14, 15–19)	All individuals in the included age group in the British Columbia COVID-19 Cohort	Longitudinal study 2019–2021	National register data	Poisson regression	Antidepressants, antipsychotics, and anxiolytics	60% increase in antidepressants and an increase of up to 47% in antipsychotics	Increase in antidepressants, antipsychotics and anxiolytics during the pandemic period (2020–2021) compared to pre-pandemic period (2019).

## Results

First, we describe the general characteristics of the studies, and then present the findings comprehensively according to the research questions. Table 1 summarises the 14 included publications, providing information on country, study population, sample size, study design and period, data source, statistical analysis, type of drug, results, and main findings.

## Characteristics of the studies

### Country

The 14 studies were conducted across 10 OECD countries, including Austria, Australia, Canada, Denmark, Israel, Italy, the United States, Portugal, Sweden and Norway. Three studies were multi-country studies. Table 1 provides full details.

### Period

Most of the included studies defined the start of the pandemic in 2020 to the end of 2021. Two studies monitored the prescription of psychotropics up to 2022 [20, 21]. The post- pandemic periods varied between two years [22, 23], three years [24–27], four years [28], five years [21], six years [29, 30], 8 years [20, 31, 32] and ten years [33].

### Age sub-groups

Six studies focused only on adolescents; four examined participants aged 10 to 19 [24, 27, 29, 31], two included an age range of 12 to 18 [22, 28]. Additionally, two studies included participants aged 0 to 19 [ 32, 30], two studies observed participants aged 0 to 18 [20, 32], and two studies focused on children and adolescents aged 0 to 17 [25, 26].

### Data sources

Most studies relied on administrative data, including national or regional health registers and clinical records. Registers provided individual-level prescription data [21, 23, 25, 26, 30, 31], while clinical records captured patient histories [22, 24, 27–29, 32], and two studies used aggregated data [20, 33].

### Psychotropic drugs

The types of psychotropic medications varied across studies, with antidepressants included in twelve studies, anxiolytics and antipsychotics each reported in eight studies, hypnotics in four studies, and stimulants in three studies.

## Overall changes in psychotropic prescribing

Across the 14 studies, changes in overall psychotropic prescribing patterns for children and adolescents during the COVID-19 pandemic were mixed. Eight studies conducted in Canada, Israel, Australia, and Sweden reported a general increase in total psychotropic medication prescriptions [21, 23, 24, 28–32]. In contrast, four studies from the USA, Canada, Australia, and Portugal indicated a decrease in prescription trends [22, 25, 27, 33]. Two studies observed fluctuations with a decline in prescriptions during the initial phase of the pandemic, followed by an increase during the second wave in 2021 [20, 26].

### Variations across psychotropic drug types

During the COVID-19 pandemic, shifts were observed in the frequency of specific types of psychotropic drug prescriptions. To some extent, most of the studies observed an increase in medication prescriptions like antidepressants, antipsychotics and particularly stimulants, whereas a few noted a significant decrease. The change estimates are shown in Table 1.

Antidepressants and anxiolytics prescriptions were particularly highlighted due to their significant increase compared to other medications [21, 24, 29, 31, 32]. Several studies highlighted substantial rises, with Bilu et al. [28] presenting a 60% increase in antidepressants in Israel and Antoniou et al. [20] finding a 21% increase in Canada. However, not all trends were upward. Two studies, one from Portugal and another from Canada, showed that the results indicated a decrease in prescriptions of antidepressants up to 50% [25] and 44% [27], respectively.

Antipsychotic prescriptions also showed varied trends in frequency. For instance, one study conducted in Canada found a 47% increase in antipsychotics [23]. Findings from Bilu et al. [ 21] particularly highlighted that an increase of 28% in antipsychotics was most pronounced among individuals with no prior psychiatric history. Findings related to hypnotics were more mixed. Some studies suggested increased use; for example, Bliddal et al. [21] reported a 39% increase, while others reported reductions or no significant change [30].

Furthermore, prescriptions for stimulants showed the most variability across studies. While some reported increases, others found significant declines [22, 28, 33]. For instance, in the United States, an 18% decrease was reported in stimulant prescriptions [33]. Similarly, reductions were also observed in Australia and Israel [22, 28]. A 48% decline in new prescriptions across three drug classes: antipsychotics, antidepressants, and stimulants, was reported in Australia [22].

Several studies showed fluctuations in prescription frequency by drug type during the pandemic. One Australian study reported a decline in antidepressant prescriptions in April 2020, followed by a steady increase in September 2020 [20]. By the end of 2022, antidepressant prescriptions had risen by 21% and antipsychotics by 13% compared to pre-pandemic levels [20]. Similarly, a multinational study observed an early decrease in anxiolytics, antidepressants, and stimulants at the start of the pandemic in 2020 in Norway and Sweden but not Italy, followed by an increase in Norway and Sweden towards the end of 2021 [26].

### Variations by gender, age and socioeconomic status

Prescription rates of psychotropics, especially stimulants and antidepressants, showed notable gender differences during the pandemic. Studies found a higher increase in new antidepressant prescriptions among girls. However, findings on stimulant prescriptions were mixed. Across countries, Kim et al. [33] found a decrease in stimulant prescriptions among boys in the United States, while Ong and Roberts [22] observed a similar trend in Australia. In contrast, Wood et al. [32] reported an increase in stimulant prescriptions in both genders in Australia. A similar rise in antipsychotic, hypnotic and anxiolytic prescriptions was noted in both genders.

Age differences in prescriptions were substantial, with adolescents experiencing a greater increase than younger children. Adolescents aged 15–19 presented an increase in antidepressant prescriptions from 24% to 40%, while school-aged children had a minor increase from 2% to 4% [24, 31]. It was also noticed that there were variations in the prescriptions of drugs by socioeconomic status. In Israel and Canada, individuals from higher socioeconomic backgrounds had higher rates of antidepressant and antipsychotic prescriptions [27, 28].

## Discussion

This scoping review synthesises 14 quantitative studies on psychotropic drug prescriptions among children and adolescents aged 0–19 across 11 OECD countries before and during the COVID-19 pandemic. These studies primarily used administrative health data to assess psychotropic drug prescriptions and covered pre-pandemic periods (2013–2019) and pandemic/post-pandemic periods (2020–2022). While total study periods ranged from 2 to 10 years, the pandemic/post-pandemic period was generally limited to short-term observations. Across all included studies, antidepressants were the most frequently examined drug class (*n* = 12), followed by anxiolytics and antipsychotics (*n* = 8 each), hypnotics (*n* = 4), and stimulants (*n* = 3). The interpretation of prescription trends across the included studies is challenged by substantial methodological heterogeneity. Pre-pandemic observation periods varied considerably in length, with only a small number of studies including extended pre-pandemic follow-up (only four studies included long pre-pandemic observation periods (> 6 years)), which may have limited the ability to accurately define underlying trends. Overall, the evidence base remains limited and heterogeneous, restricting firm conclusions. This review therefore provides an early synthesis to map current evidence and identify key gaps.

The overall findings indicated a general increase in prescriptions during the pandemic. Antidepressants showed an increase in use, while trends for stimulants were mixed. At the individual level, this may reflect the heightened mental health challenges faced by children and adolescents during the COVID-19 pandemic [5, 7]. Disruptions to daily life, social isolation, and uncertainties about the future became widespread during this period [3, 34]. Adolescents might be more susceptible to social isolation from peers than younger children [6]. Additionally, the increased academic demands and stricter schoolwork requirements may have been challenging for this age group [6]. Conversely, certain vulnerable youths, such as those diagnosed with autism or ADHD, may have encountered less stress from the educational environment. This may have helped improve mental health [35]. From a family perspective, the COVID-19 pandemic likely impacted the home environment in various ways [6]. Disruptions caused by the pandemic, such as school closures and limited access to in-person mental health services, may have contributed to increased stress levels among families [6, 35]. These factors may have contributed to the onset of symptoms related to anxiety and depression as an acute response during crises, which sometimes might not meet the criteria for a formal diagnosis [23].

Furthermore, variability in the frequency and types of psychotropic drugs prescribed was identified. Regardless of the observation period, a consistent increase in antidepressant prescriptions was observed [21, 24, 31, 32], likely driven by rising anxiety and depression linked to isolation from networks beyond the nuclear family and disrupted daily routines [8, 36]. This increase may also reflect a shift toward pharmacological treatment as access to face-to-face therapy was limited, along with a reduction in stigma around mental health [35]. Additionally, antidepressants, particularly SSRIs and SNRIs, are often the first-line treatment for many anxiety disorders, including generalised anxiety disorder, social anxiety disorder, and panic disorders [2]. Notably, antidepressants were the most frequently examined drug class in the included studies (*n* = 12), which may partly explain the greater consistency in findings compared to other drug categories. In contrast, trends varied more widely across other drug classes: while anxiolytic prescriptions also generally increased, antipsychotic and hypnotic prescriptions showed mixed patterns, and stimulant use exhibited the most substantial variability, ranging from significant increases to marked declines [22, 23, 28, 30, 33]. These inconsistencies may be attributed to country-specific responses to the pandemic, disruptions in care delivery, changes in clinical practice, or specific clinical reactions to the context of each diagnosis [11].

The timing of assessments is crucial when interpreting prescription trends. Moreover, most studies defined the pandemic’s onset broadly, spanning from early 2020 to the end of 2021. Such variation tends to be particularly problematic for interrupted time-series analyses, as the timing and intensity of restrictions differed markedly across countries and over time. Early declines, such as those reported in Australia [22] and Portugal [25] might result from a lack of governmental strategies to deal with crises and increased health care demands [25]. Ong and Roberts [22] suggest that the pandemic’s context more likely influenced these changes than actual mental health trends. In contrast, later studies may show different patterns as healthcare systems adjusted and alternative pathways for care and treatment follow-up became more accessible. However, the COVID-19 pandemic is still a relatively recent public health crisis. Its broader social and health system impacts may disproportionately affect vulnerable populations [37]. The long-term consequences of the pandemic on mental health and psychotropic prescribing trends among children and adolescents remain unclear. Additionally, it is not yet fully understood whether the observed changes reflect temporary pandemic-related disruptions or more persistent shifts in mental health needs and prescribing patterns. Longer follow-up periods and continued pharmacoepidemiological monitoring are therefore needed to distinguish short-term effects from longer-term trends and the aftermath of the pandemic in such trends [37].

The gender differences shown in this review align with the broader literature describing psychiatric gender-specificities [38]. One might hypothesise that such gendered patterns were exacerbated or clearer in the context of the pandemic [21, 39]. Prescription of antidepressants was particularly pronounced among girls without a documented psychiatric history during the pandemic [28]. In contrast, studies on stimulant prescriptions, more commonly associated with boys, showed variation across countries. In the USA, a decrease in prescriptions was suggested to be connected to school closures and potentially reduced need for ADHD symptom management [33]. In contrast, an Australian study reported an increase, potentially due to limited access to non-pharmacological interventions and greater recognition of symptoms during the pandemic [32].

More studies evaluated psychotropic drug prescriptions among older adolescents [20, 21, 23, 24, 26, 31], whereas data for children younger than 9 years old were limited to one study [23]. This may reflect the non-usual practice of psychotropic drugs in children within this age group [23] even though the prescription of psychotropic medication for older adolescents is a relatively new practice in which the pros and cons have been constantly discussed [20, 21, 23, 24, 26, 31], in the combination and/or isolation of other therapeutic alternatives [32].

Socioeconomic disparities were analysed and observed in two of the studies included in this scoping review [27, 28]. Psychotropic drug use may be shaped not only by clinical need but also by socioeconomic conditions, welfare structures and access to care [5]. Children from more advantaged backgrounds seemed to have higher rates of psychotropic medication prescription in comparison to those from less advantageous backgrounds [27, 28]. From a health inequality perspective, this finding corroborates with pre-existing literature that indicates stark inequalities in mental health among adolescents, i.e., those in the most disadvantaged circumstances tend to have more psychiatric issues (more in need). The pandemic may strengthen existing inequalities [6]. However, higher prescribing rates among more advantaged groups do not necessarily reflect greater need, but may instead indicate differential access to mental health services, particularly during crises when disadvantaged groups face greater barriers to care [6, 36]. During the pandemic, access to healthcare services, including mental health care and prescription medications, was more readily available to families with greater resources, potentially exacerbating existing health inequities [5]. In addition, socioeconomic position influences help-seeking behaviour, suggesting that prescription patterns may reflect differences in healthcare utilisation rather than underlying need [27, 28].

Cross-country comparisons underscore the role of contextual and temporal factors in interpreting psychotropic prescription trends. A multi-country study reported initial declines in Norway and Sweden, followed by increases in all three countries examined (including Italy), reflecting evolving healthcare responses [26]. Denmark reported a sharper increase in antidepressant use than other Nordic countries [30]. Overall, the rates were higher in the Nordic region than in Southern Europe, likely reflecting differences in healthcare systems, with Nordic countries having universal, tax-funded care, and Southern Europe relying more on mixed public–private models [19]. Only Denmark [21] and Canada [20] covered both pandemic waves, both reporting increased prescriptions post-pandemic. National responses likely shaped these trends; for instance, extended school closures in Canada [20] may have worsened youth mental health, while limited care access in Australia [22, 29, 32] may have delayed treatment. Study design also mattered: population-based studies [23, 25, 29, 33] provided broader insights, whereas smaller studies [22, 24] had limited generalisability. Furthermore, differences in pandemic strategies across countries could be linked to broader welfare state models and socio-political contexts. This may indicate that prescribing trends reflect structural differences and how national policy responses impacted healthcare access during the pandemic [40].

### Strengths and limitations

This study’s strengths include a comprehensive search across five databases, adherence to international scoping review guidelines [16], and use of the PE(C)O-model [41] to focus the research question and strategy. Two independent reviewers enhanced methodological rigor. However, limitations exist. The exclusion of non-English publications and grey literature may have led to omitting relevant studies. Furthermore, some studies included long pre-pandemic observation periods, follow-up during the pandemic and post-pandemic periods was generally short. This might limit the ability to differentiate between short-term and long-term changes in psychotropic drug prescriptions. Additionally, the included studies did not consistently distinguish between the prevalence and incidence of psychotropic drug prescriptions. As a consequence, observed changes might reflect both changes in ongoing treatments and new (treatment) prescriptions. The substantial variation in study design, observation periods, and analytical approaches further limits the generalisability of findings across studies. Since the review focused on children and adolescents aged 0–19, most included studies focused on adolescents. This may limit the generalisability of the findings to younger children, for whom prescription patterns may differ. Additionally, the focus on OECD countries limits the generalisability of the findings to low and middle-income settings, which may have different healthcare systems and cultural practices influencing psychotropic medication use [16]. Taken together, these limitations indicate that the findings should be interpreted with caution and primarily as indicative rather than conclusive.

### Implications for policy, practice and future research

This scoping review highlights the pandemic’s potential impact on psychotropic prescribing among children and adolescents. The observed increases, particularly in antidepressants, may reflect underlying mental health needs and/or changes in healthcare access and treatment practices during the pandemic. At this stage, the value of this review lies in systematically synthesising a fragmented body of evidence and identifying emerging patterns and inconsistencies, thereby helping to guide future research priorities rather than providing definitive answers. These trends raise important questions about the management of mental healthcare and the role of psychotropic medication as a crisis response strategy. Strengthening preparedness and resilience across health, social, political, and climate-related crises is essential to ensure equitable and adequate mental health support. Future research should include high-quality longitudinal studies to assess whether current prescribing trends persist beyond the pandemic. Further work is also needed to explore subgroup differences (e.g., by age, sex, and socioeconomic status) and to examine how contextual factors influence prescribing patterns. In this context, the present review serves as a starting point and highlights the importance of continued monitoring of pharmacoepidemiological data and ongoing research in this field.

## Conclusion

This scoping review suggests an overall pattern of increased psychotropic drug prescriptions for children and adolescents across several OECD countries during the pandemic. However, observed trends varied widely across drug types, population subgroups, countries, and study designs. In addition, follow-up periods ranged from 1 to 3 years, reducing comparability across studies and complicating the interpretation of the short- versus longer-term effects of the pandemic. These differences should be taken into account when interpreting overall patterns. Given the substantial methodological heterogeneity and variability in observation periods, the generalisability of these findings should be approached with caution. Further high-quality research is still needed to determine the long-term implications of the pandemic on psychotropic drug prescription and to inform future mental healthcare strategies in the context of crisis preparedness.

## Supplementary Information

Supplementary Material 1

Supplementary Material 2

Supplementary Material 3

Supplementary Material 4

## Data Availability

Not applicable.
