# Supplementary material for: The COVID-19 pandemic’s impact on psychotropic medication prescriptions for children and adolescents: a scoping review of quantitative longitudinal studies
Source: Ann Gen Psychiatry. 2026 Aug 17;25:70. doi: 10.1186/s12991-026-00692-z (PMC13480077; doi:10.1186/s12991-026-00692-z)
Supplement: Supplementary file 1 [file 12991_2026_692_MOESM1_ESM.docx]

# Supplementary file 1: Search strategy

P: Children and adolescents (block search 1)

E: Covid-19 pandemic (block search 2)

O: Psychotropic drugs (block search 3)

**Database: PubMed 203 articles**

| Search number | Search |  |
| --- | --- | --- |
| 1 | "Adolescent" OR "Child" | Mesh-terms |
| 2 | Child OR Adole* OR Infant OR Teen OR Youth OR Kid OR Tween OR Young OR “School age” | Title/ Abstract |
| 3 | 1 OR 2 |  |
| 4 | “COVID-19” | Mesh-terms |
| 5 | COVID-19 OR Pandemic OR Corona virus OR COVID OR covid19 OR covid2019 | Title/ abstract |
| 6 | 4 OR 5 |  |
| 7 | "Psychotropic Drugs” OR prescriptions "Antidepressive Agents" OR "Hypnotics and Sedatives" OR "Anti-Anxiety Agents" OR anxiolytics OR  “Antipsychotic drugs” OR “Central stimulants” | Mesh-terms |
| 8 | Psychotropic* OR psychotropic prescription OR Psychotropic use OR Antidepressant* OR “Mood stabilizers” OR Hypnotic* OR “Hypnotic medication” OR “Sleep aid” OR Anxiolytic* OR “Anti-anxiety medication” OR “ADHD medication” OR “Attention deficit hyperactivity disorder medication” OR Stimulant* | Title/abstract |
| 9 | 7 OR 8 |  |
| 10 | 3 AND 6 AND 9 |  |

**Database: CINAHL 61 articles**

| Search number | Search |  |
| --- | --- | --- |
| 1 | "Adolescence" OR "Child" | Mesh-terms (subject headings) |
| 2 | Child OR Adole* OR Infant OR Teen OR Youth OR Kid OR Tween OR Young OR “School age*” | Title/ Abstract |
| 3 | 1 OR 2 |  |
| 4 | “COVID-19” OR “COVID-19 Pandemic” OR SARS-CoV-2 | Mesh-terms (subject headings) |
| 5 | COVID-19 OR Pandemic OR “Corona virus” OR COVID OR covid19 OR covid2019 | Title/ abstract |
| 6 | 4 OR 5 |  |
| 7 | "Psychotropic Drugs" OR "Prescriptions" OR "Antidepressive Agents" OR "Hypnotic and Sedative" OR "Antianxiety Agents" | Mesh-terms (subject headings) |
| 8 | Psychotropic* OR psychotropic prescription OR Psychotropic use OR Antidepressant* OR “Mood stabilizers” OR Hypnotic* OR “Hypnotic medication” OR “Sleep aid” OR Anxiolytic* OR “Anti-anxiety medication” OR “ADHD medication” OR “Attention deficit hyperactivity disorder medication” OR Stimulant* | Title/abstract |
| 9 | 7 OR 8 |  |
| 10 | 3 AND 6 AND 9 |  |

**Database: Web Of Science 505 articles**

| Search number | Search |  |
| --- | --- | --- |
| 1 | "Adolescence" OR "Child" | Mesh-terms (subject headings) |
| 2 | Child* OR Adolescen* OR Infant* OR Teen* OR Youth* OR Kid* OR Tween* OR Young* OR “School age*” | Title/ Abstract |
| 3 | 1 OR 2 |  |
| 4 | “COVID-19” OR “COVID-19 Pandemic” OR SARS-CoV-2 | Mesh-terms (subject headings) |
| 5 | COVID-19 OR Pandemic OR “Corona virus” OR COVID OR covid19 OR covid2019 | Title/ abstract |
| 6 | 4 OR 5 |  |
| 7 | "Psychotropic Drugs" OR "Prescriptions" OR "Antidepressive Agents" OR "Hypnotic and Sedative" OR "Antianxiety Agents" OR Antipsychotic Agents” | Mesh-terms (subject headings) |
| 8 | Psychotropic* OR psychotropic prescription OR Psychotropic use OR Antidepressant* OR “Mood stabilizers” OR Hypnotic* OR “Hypnotic medication” OR “Sleep aid” OR Anxiolytic* OR “Anti-anxiety medication” OR “ADHD medication” OR “Attention deficit hyperactivity disorder medication” OR Stimulant* | Title/abstract |
| 9 | 7 OR 8 |  |
| 10 | 3 AND 6 AND 9 |  |

**Database: Scopus 20 articles**

| Search number | Search |  |
| --- | --- | --- |
| 1 | "Adolescent" OR "Child" | Mesh-terms |
| 2 | Child* OR Adolescen* OR Infant* OR Teen* OR Youth* OR Kid* OR Tween* OR Young* OR “School age*” | Title/ Abstract |
| 3 | 1 OR 2 |  |
| 4 | “Coronavirus Infections” OR  “COVID-19” OR Pandemic* OR SARS-CoV-2 | Mesh-terms |
| 5 | COVID-19 OR Pandemic OR Corona virus OR COVID OR covid19 OR covid2019 | Title/ abstract |
| 6 | 4 OR 5 |  |
| 7 | "Psychotropic Drugs" OR "Prescriptions" OR "Antidepressive Agents" OR "Hypnotics and Sedatives" OR "Anti-Anxiety Agents" OR  "Attention Deficit Disorder with Hyperactivity medication" OR “stimulants” | Mesh-terms |
| 8 | Psychotropic* OR psychotropic prescription OR Psychotropic use OR Antidepressant* OR “Mood stabilizers” OR Hypnotic* OR “Hypnotic medication” OR “Sleep aid” OR Anxiolytic* OR “Anti-anxiety medication” OR “ADHD medication” OR “Attention deficit hyperactivity disorder medication” OR Stimulant* | Title/abstract |
| 9 | 7 OR 8 |  |
| 10 | 3 AND 6 AND 9 |  |

**Database: PsycINFO 73 articles**

| Search number | Search |  |
| --- | --- | --- |
| 1 | "Adolescent" OR "Child" | Mesh-terms |
| 2 | Child* OR Adole* OR Infant* OR Teen* OR Youth OR Kid* OR Tween OR Young OR “School age*” | Title/ Abstract |
| 3 | 1 OR 2 |  |
| 4 | “COVID-19” | Mesh-terms |
| 5 | COVID-19 OR Pandemic OR Corona virus OR COVID OR covid19 OR covid2019 | Title/ abstract |
| 6 | 4 OR 5 |  |
| 7 | “Psychotropic drugs” OR “Hypnotic drug” OR “Anxiolytic drug” OR “Antianxiety drug” OR “Antidepressant drug” OR “Sedatives” | Mesh-terms |
| 8 | Psychotropic* OR psychotropic prescription OR Psychotropic use OR Antidepressant* OR “Mood stabilizers” OR Hypnotic* OR “Hypnotic medication” OR “Sleep aid” OR Anxiolytic* OR “Anti-anxiety medication” OR “ADHD medication” OR “Attention deficit hyperactivity disorder medication” OR Stimulant* | Title/abstract |
| 9 | 7 OR 8 |  |
| 10 | 3 AND 6 AND 9 |  |
