# Supplementary material for: The COVID-19 pandemic’s impact on psychotropic medication prescriptions for children and adolescents: a scoping review of quantitative longitudinal studies
Source: Ann Gen Psychiatry. 2026 Aug 17;25:70. doi: 10.1186/s12991-026-00692-z (PMC13480077; doi:10.1186/s12991-026-00692-z)
Supplement: Supplementary file 2 [file 12991_2026_692_MOESM2_ESM.docx]

Supplementary file 2: data extraction table of the included literature.

| Authors and publication year | Country | Aim and hypothesis | Study population | Sample size | Study design and period | Data source | Statistical analysis | Type of drugs | Other variables | Frequency of change | Main Findings | Type of healthcare | Demographic factors |
| --- | --- | --- | --- | --- | --- | --- | --- | --- | --- | --- | --- | --- | --- |
| Antoniou et al. 2023 | Canada | The aim was to examine trends in prescriptions of antidepressants and antipsychotics among children and adolescents during covid-19 | 0 to 18 (0-13, 14-18) | All residents of Ontario, Canada the years the study was conducted | Longitudinal  2014- 2022 | Register data at regional level | Interrupted time series analysis | Antidepressants, antipsychotics | No. | 21,4% increase in antidepressants  13,2% antipsychotics | Initial decrease in antidepressant prescriptions and then increased in both psychotropics during the pandemic period (2020-2022) compared to the pre-pandemic period (2014-2020). | Universal health care | Stratified by age and sex |
| Bilu et al. 2023 | Israel | To examine if the impact of covid-19 on adolescents health including prescriptions | 12 to 17 | N= 200,000 | Longitudinal  2017- 2021 | Administrative clinical data at the individual level | Regression models | Antidepressants, anxiolytics, antipsychotics, stimulants | Psychiatric diagnoses | 25% increase of antidepressants, 28% increase of antipsychotics, 10% decrease of stimulants | Increase in antidepressants and antipsychotics, decrease in stimulants during the pandemic (2020-2021) compared to pre-pandemic period (2017-2019). | Universal health care | Age, sex, SES i.e., ethnicity, geographic area, urban vs rural |
| Bliddal et al 2023 | Denmark | To examine if the COVID-19 pandemic is associated with changes in psychotropic medication use or rates of diagnosed psychiatric disorders in youths? | 5 to 17  (5-11, 12-17) | All residents of Denmark, the years the study was conducted | Longitudinal  2017- 2022 | Register data at national level | Regression models | Antipsychotics, antidepressants, anxiolytics, hypnotics | Psychiatric diagnoses | Increased with 39% of hypnotics, 13% of antidepressants and 19% of stimulants | Increase in all psychotropic drugs during the pandemic period (2020-2022) compared to the pre-pandemic period (2017-2020). | Universal health care | Stratified by age and sex |
| Daneshmands et al. 2023 | Canada | The aim was to assess changes in primary healthcare utilization related to depression and anxiety among children and youth | 10 to 19 (10-14, 15-19) | N= 3974 | Longitudinal  2018- 2021 | Administrative clinical data at the individual level | Interrupted time series analysis and regression models | Antidepressants | Healthcare visits | 33,5% increase of antidepressants | Increase in antidepressants for both children and school-age children during the pandemic (2020-2021) compared to the pre-pandemic period (2018-2020). | Universal primary health care | Stratified by age and sex |
| De Oliveira Costa et al. 2022 | Australia New Zeeland | The aim was to describe recent patterns of antidepressant use in Australia, both prior to and during the first year of the COVID-19 pandemic covering the period 2015–2021. | 10 to 17 | All residents of Australia during the years the study was conducted | Longitudinal  2015- 2021 | Administrative clinical data at the individual level | Interrupted time series analysis | Antidepressants | No | Increase of 56,9% in new use of antidepressants for girls and 45,75 for boys.  Prevalent use: increase of 27,2% for girls and 34% for boys | Increase in antidepressants during the pandemic period (2020- 2021), with a higher increase in new use among girls than boys compared to the pre-pandemic period (2015-2019). | Universal health care | Stratified by age and sex |
| Estrela et al. 2021 | Portugal | The aim was to assess the trends of psychotropic drugs and the impact of covid-19 | 0 to 17 (0-7, 8-17) | All residents of Portugal, the years the study was conducted | Longitudinal  2018-2021 | National register data | Interrupted time series analysis | Antidepressants, anxiolytics, hypnotics | No | Decrease of 50% of antidepressants, hypnotics and anxiolytics | Overall decrease of antidepressants and anxiolytics, with a higher decrease of antidepressants during the pandemic period (2020- beginning of 2021) compared to the pre-pandemic period 2018-2020). | Universal health care | Stratified by age and sex |
| Kim et al. 2023 | U. S | The aim was to investigate trends of dispensed stimulant medications in relation to the COVID-19 pandemic response | 0 to 18 (0-12, 13-18) | All residents of Massachusetts, USA the years the study was conducted | Longitudinal  2011-2021 | Aggregated register data at regional level | Interrupted time series analysis | Stimulants | No | Decrease of 18.4% of stimulants among boys and 13% among girls | Decrease in stimulant prescriptions during the pandemic period (2020-2021), with a higher decrease for boys than girls compared to the pre-pandemic period (2011-2019). | Universal health care | Stratified by age and sex |
| Ong & Roberts, 2022 | Australia | The aim was to examine the effects of the COVID-19 pandemic and rapid introduction of telehealth consultations on the prescribing trends and medication change | 12 to 18 | N= 5960 | Longitudinal    2019-2021 | Administrative clinical data at the individual level | Regression models | Antipsychotics, antidepressants, stimulants | Telehealth consultations | 48,4% decrease of new prescriptions and 15,1% of follow-up prescriptions | Decrease in prescriptions of antipsychotics, antidepressants and stimulants, including new and follow-up prescriptions during pandemic period (2020- 2021) compared to the pre-pandemic period (2019-2020). | Universal primary care | Stratified by age and sex |
| Otter et al 2024 | Austria | The aim was to investigate the influence of the COVID-19  pandemic on prescription patterns of common psychotropic drugs with a specific focus on adolescents | 10 to 19 (10-14, 15-19) | N= 850,000 | Longitudinal  2013-2021 | Register data | Interrupted time series analyses | Antidepressants, antipsychotics | No | Antidepressants:  Age 15 to 19:  Increase of 1.49% for females  Increase of 2.04% for males  Age 10 to 14:  Increase of 104% compared to 35% among boys aged 10 to 14 years old.  Antipsychotics:  Increase of 5.38% for females  Increase of 5.61% for males | Increase in prescriptions of antidepressants and antipsychotics among adolescents, particularly pronounced in girls aged 10- 14 during the pandemic period (2020-2021) compared to the pre-pandemic period (2013- 2020). | Universal health care | Stratified by age and sex |
| Pedersen et al. 2023 | Norway  Sweden  Italy | The aim was to explore how the COVID-19 pandemic affected drug utilisation in children and adolescents in Norway, Sweden, and Italy, by child age. | 0 to 17 (0-5, 6-11, 12-17)  0-14 in Italy | N= 3,455,521  (Total)  N=136,188 Italy  N= 1,160,431 Norway  N= 2,158,902 Sweden | Longitudinal  2018-2021 | National and regional register data | Interrupted time series analysis | Antidepressants, antipsychotics, anxiolytics, hypnotics, stimulants | Healthcare visits | Decrease of 2.5 per 1000 stimulant prescriptions, a decrease of 0.7 per 1000 antidepressant prescriptions and a decrease of 0.6 per 1000 prescriptions of anxiolytics | Initial decrease in all prescriptions and then an increase in antidepressants, stimulants and anxiolytics primarily in Sweden and Norway during the pandemic period (2020-2021) compared to the pre-pandemic period (2018- 2020). | Primary care/ outpatient care | Maternal education level, household composition |
| Stephenson et al. 2022 | Canada | The aim of the study was to determine whether the rates of patients presenting with or being treated for anxiety and/or depression from March to December 2020 differed from expected rates based on pre-COVID-19 pandemic patterns. | 10 to 18 | N= 25,695 (7,96% of total) | Longitudinal study  2018- 2021 | Administrative clinical data at the individual level | Regression models | Antidepressants | Healthcare visits | Overall, 44% decrease in antidepressants | Decrease in antidepressants during the pandemic period (2020- 2021) compared to pre-pandemic period (2018-2020). | Primary care / family doctor | Income quintile, geographic distribution (population was from an area of high rates of covid-19) |
| Tiger et al. 2023 | Sweden, Norway, Denmark | The aim was to study patterns of antidepressant, anxiolytic, and hypnotic drug utilization in Denmark, Norway, and Sweden during the first year of the COVID-19 pandemic. | 0 to 19 | Denmark n= 1,309,228 Norway n=1,259,752 Sweden  n=2,289,467 (2015-2019)  Denmark n=1,296,400 Norway n=1,248,749 Sweden  n=2,403,730 (2020) | Longitudinal study  2015 – 2021 | National register data | Regression models | Antidepressants, hypnotics, anxiolytics | No | Sweden; 21,4% increase of antidepressants, 9,5% increase of antipsychotics  Norway: 17,4% increase of antidepressants  Denmark: 31,3% increase in antidepressants | Increase in antidepressants and antipsychotics during the pandemic period (2020- 2021) compared to the pre-pandemic period (2015- 2019. | Universal primary care | Stratified by age and sex |
| Wood et al. 2023 | Australia | The aim of the study was to investigate the longitudinal patterns of dispensing psychotropic medications to children and adolescents in Australia from 2013 to 2021 | 0 to 18 (0-6, 7-12, 13-18) | N= 32,351 | Longitudinal  2013-2021 | Administrative clinical data at the individual level | Regression models | Antidepressants, antipsychotics, anxiolytics, hypnotics, stimulants | No | Antidepressants: Boys: Increased by 6.1% Girls: Increased by 22.2% Stimulants: Boys: Increased by 14.5% Girls: Increased by 27.7% | Primarily an increase in antidepressants and stimulants during the pandemic period (2020- 2021) compared to the pre-pandemic period (2013-2020). | Universal primary care/ psychiatrist | Stratified by age and sex |
| Zandy et al. 2023 | Canada | The aim was to examine mental health-related healthcare  service utilisation patterns and psychotropic drug  dispensations in British Columbia, Canada | 0 to 19  (0-5, 5-9, 10-14,  15-19) | All individuals in the included age group in British Columbia COVID-19 Cohort | Longitudinal study  2019- 2021 | National register data | Regression models | Antidepressants, antipsychotics, anxiolytics | Outpatient visits, emergency department visits, hospital admissions | 60% increase in antidepressants and an increase of up to 47% in antipsychotics | Increase in antidepressants, antipsychotics and anxiolytics during the pandemic period (2020 – 2021) compared to pre-pandemic period (2019). | Universal | Stratified by age and sex |
