# Supplementary material for: The COVID-19 pandemic’s impact on psychotropic medication prescriptions for children and adolescents: a scoping review of quantitative longitudinal studies
Source: Ann Gen Psychiatry. 2026 Aug 17;25:70. doi: 10.1186/s12991-026-00692-z (PMC13480077; doi:10.1186/s12991-026-00692-z)
Supplement: Supplementary file 4 [file 12991_2026_692_MOESM4_ESM.docx]

Supplementary file 3: *OECD Member Countries (2025)*

| Australia | Austria | Belgium | Canada |
| --- | --- | --- | --- |
| Chile | Colombia | Costa Rica | Czech Republic |
| Denmark | Estonia | Finland | France |
| Germany | Greece | Hungary | Iceland |
| Ireland | Israel | Italy | Japan |
| Korea, Republic of (South Korea) | Latvia | Lithuania | Luxembourg |
| Mexico | Netherlands | New Zealand | Norway |
| Poland | Portugal | Slovakia | Slovenia |
| Spain | Sweden | Switzerland | Turkey |
| United Kingdom | United States |  |  |
